# Monocytes Induce STAT3 Activation in Human Mesenchymal Stem Cells to Promote Osteoblast Formation

**DOI:** 10.1371/journal.pone.0039871

**Published:** 2012-07-03

**Authors:** Vicky Nicolaidou, Mei Mei Wong, Andia N. Redpath, Adel Ersek, Dilair F. Baban, Lynn M. Williams, Andrew P. Cope, Nicole J. Horwood

**Affiliations:** 1 Kennedy Institute of Rheumatology, Nuffield Department of Orthopaedics, Rheumatology and Musculoskeletal Sciences, University of Oxford, London, United Kingdom; 2 The Wellcome Trust Centre for Human Genetics, University of Oxford, Oxford, United Kingdom; 3 Centre for Molecular and Cellular Biology of Inflammation, Division of Immunology, Infection and Inflammatory Diseases, Academic Department of Rheumatology, King's College School of Medicine, London, United Kingdom; University of Sao Paulo - USP, Brazil

## Abstract

A major therapeutic challenge is how to replace bone once it is lost. Bone loss is a characteristic of chronic inflammatory and degenerative diseases such as rheumatoid arthritis and osteoporosis. Cells and cytokines of the immune system are known to regulate bone turnover by controlling the differentiation and activity of osteoclasts, the bone resorbing cells. However, less is known about the regulation of osteoblasts (OB), the bone forming cells. This study aimed to investigate whether immune cells also regulate OB differentiation. Using *in vitro* cell cultures of human bone marrow-derived mesenchymal stem cells (MSC), it was shown that monocytes/macrophages potently induced MSC differentiation into OBs. This was evident by increased alkaline phosphatase (ALP) after 7 days and the formation of mineralised bone nodules at 21 days. This monocyte-induced osteogenic effect was mediated by cell contact with MSCs leading to the production of soluble factor(s) by the monocytes. As a consequence of these interactions we observed a rapid activation of STAT3 in the MSCs. Gene profiling of STAT3 constitutively active (STAT3C) infected MSCs using Illumina whole human genome arrays showed that Runx2 and ALP were up-regulated whilst DKK1 was down-regulated in response to STAT3 signalling. STAT3C also led to the up-regulation of the oncostatin M (OSM) and LIF receptors. In the co-cultures, OSM that was produced by monocytes activated STAT3 in MSCs, and neutralising antibodies to OSM reduced ALP by 50%. These data indicate that OSM, in conjunction with other mediators, can drive MSC differentiation into OB. This study establishes a role for monocyte/macrophages as critical regulators of osteogenic differentiation via OSM production and the induction of STAT3 signalling in MSCs. Inducing the local activation of STAT3 in bone cells may be a valuable tool to increase bone formation in osteoporosis and arthritis, and in localised bone remodelling during fracture repair.

## Introduction

Fragility fractures as a consequence of osteoporosis, as well as bone loss associated with chronic inflammatory diseases such as inflammatory arthritis, are extremely common and represent major unmet clinical problems. Over 200 million people suffer from osteoporosis worldwide, and 9 million fragility fractures occur every year [1], with the most severe often resulting in permanent disability and increased mortality rates. To reduce the socioeconomic impact of these common clinical conditions, it is imperative that ways to promote bone formation are discovered. However, treatment options remain inadequate or non-existent. Although the inhibition of bone resorbing osteoclasts (OCs) using drugs such as bisphosphonates, calcitonin and selective estrogen receptor modulators will prevent further bone loss, these agents do not stimulate new bone formation. In terms of bone anabolic factors, parathyroid hormone increases bone mass when administered intermittently but can only be given to patients for a limited number of years. Strontium ranelate has also been shown to have bone anabolic effects but it is only approved for use in Europe [2]. In fracture repair, recombinant forms of bone morphogenetic protein 2 (BMP2) and BMP7 have been given locally, but cannot be used systemically and their efficacy has not been proven as animal models would have indicated [3], [4]. Anti-sclerostin antibodies have shown early promise but are currently limited to systemic use and face potential safety issues, including carcinogenesis [5]. Thus, there is a significant unmet need for the identification and characterization of novel bone anabolic agents.

Understanding the molecular and cellular details of the pathways that control bone formation is critical for the discovery of new bone anabolics. Osteoblasts (OB) are derived from mesenchymal stem cells (MSC), a type of adult stem cell that resides in the bone marrow [6], [7], [8]. Osteogenic differentiation of MSCs is under the control of OB-specific transcription factors such as Runx2 and Osterix [9], [10], [11], as well as numerous secreted factors of paracrine, autocrine, and endocrine origin. These include certain BMPs, PTH, FGF, IGF, endothelin-1, and prostaglandin agonists [12], [13], [14]. Canonical Wnt signalling also regulates OB differentiation [15], [16] and cooperates with Runx2 and Osterix to maintain and promote OB maturation. Dickkopf-1 (Dkk1) and sclerostin, an exclusively osteocyte-derived inhibitor of osteoblastogenesis, have been shown to block Wnt signaling [17], [18]. Dkk1-specific antibodies reversed bone destruction in a mouse model of rheumatoid arthritis [19], while antibodies to sclerostin are currently under investigation for the treatment of osteoporosis and fracture healing in clinical trials [20].

Whilst these current bone anabolic agents target known pathways associated with OB differentiation, a novel approach would be to exploit the more recent understanding of the regulatory role of the immune system in bone biology. Since the early observations that persistence of inflammation leads to bone loss, multiple immune components including T cells, B cells and various cytokines, have been shown to regulate the OCs and OBs [21], [22], [23], [24]. More recently, it has been shown that resident macrophages, termed OsteoMacs, are intercalated within murine and human osteal tissues and are required for osteogenesis [25]. These macrophages are intimately associated with regions of bone modeling *in situ* whilst their depletion caused a complete loss of the OB bone-forming surface at this modeling site. The same authors have also shown that macrophages are involved in intramembranous fracture repair [26]. These findings suggest that macrophages can regulate both the differentiation and mineralization of OBs but the mechanisms and mediators governing these interactions remain unknown.

Various aspects of the interactions between MSCs and immune cells have been investigated at length, particularly those involved in the regulatory role of MSCs on haematopoiesis in the bone marrow niche [27], [28], [29], as well as those involved in conferring the immunosuppressive properties of MSCs. Several groups have suggested that the molecular mechanisms involved in the latter require both cell-cell interactions as well as soluble factors [30], [31], [32], [33] including interleukin-10 (IL-10) [34], IL-1β and TGFβ [35], IFNγ and TNFα [36] and PGE_2_ [37], [38]. All of these studies have focused on the ability of MSCs to limit the proliferation of immune cells; however these relationships are likely to be reciprocal and the influence of immune cells on MSC lineage commitment, and hence the skeletal system are largely unreported. We hypothesized that the mechanisms and factors involved in immune suppression also affects MSC differentiation and/or function. Indeed, IL-1β and TGFβ have been shown to promote the osteogenic differentiation of MSCs [39], [40].

We report here that monocyte/macrophages can directly promote MSC osteogenic differentiation through cell contact interactions, thus resulting in the production of osteogenic factors by the monocytes. This mechanism is mediated by the activation of STAT3 signaling pathway in the MSCs that leads to the up-regulation of OB-associated genes such as Runx2 and alkaline phosphatase (ALP), and the down-regulation of inhibitors such as DKK1 to drive the differentiation of MSCs into OBs. Additionally, the up-regulation of Oncostatin (OSM) receptor (OSMR) and Leukemia Inhibitory Factor receptor (LIFR) following STAT3 activation in MSCs, indicated OSM as a key monocyte-derived osteogenic factor acting to enhance osteogenic differentiation via the STAT3 signaling pathway. These findings further our understanding of cellular interactions underlying bone formation to provide therapeutic targets for the next generation of bone anabolic factors.

## Results

### Monocytes are Potent Inducers of Osteogenic Differentiation

**Figure 1:**
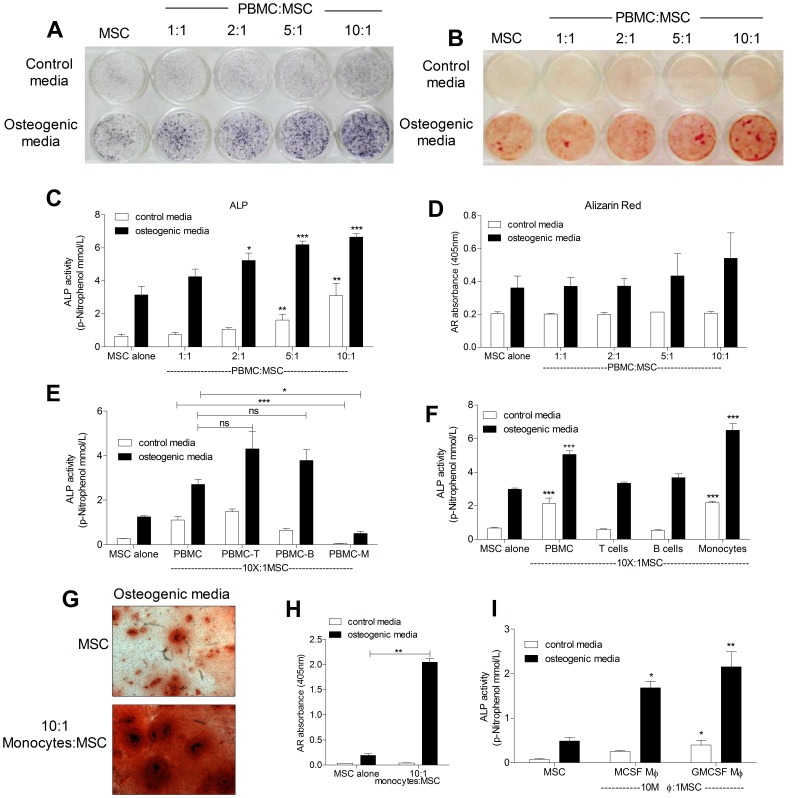
Monocytes potently induce osteogenic differentiation of MSCs. 1×10^4^ MSCs were cultured either alone or with increasing numbers of PBMCs (1,2,5 or 10×10^5^ PBMCs), in the presence or absence of osteogenic stimuli. After 7 days, MSC ALP was assessed by staining (A) and after 21 days, bone nodule formation was visualised by Alizarin Red S staining (B). ALP activity (C) and Alizarin Red content (D) were also quantified. Populations of T cells, B cells or monocytes within PBMCs were either depleted (E) or enriched (F) prior to culture with MSC (at 10∶1 ratio) and ALP activity was quantified after 7 days. Co-cultures of MSCs with enriched monocyte populations were also kept for 21 days in osteogenic media when bone nodule formation was assessed using Alizarin Red S staining (G) and quantification of dye content (H). Monocytes were differentiated to macrophages (Mφ) in either M-CSF or GM-CSF for 5 days prior to co-culture with MSCs at a ratio of 10∶1. After 7 days of co-culture, ALP activity was quantified (I). Phase contrast pictures (10X) are representative of three independent experiments performed. Graphs show means ± SEM of three independent experiments performed in triplicates. *p≤0.05, **p≤0.01, ***p≤0.001.

To ascertain the effect of immune cells on OB differentiation, co-cultures of human MSCs and peripheral blood mononuclear cells (PBMCs) isolated from healthy human donors were established at increasing ratios of PBMC:MSC. ALP staining and quantification of enzyme activity revealed that co-culture in osteogenic medium resulted in a dose dependent increase in ALP-positive cells as compared to mono-cultures of MSCs (Figure 1A and 1C). Likewise, ALP activity was significantly elevated in co-cultures in control medium compared to MSCs alone although the magnitude was lower than that observed in the presence of osteogenic medium (Figure 1A and 1C). The ability of PBMCs to induce osteogenesis was confirmed in bone nodule formation assays, as visualized by alizarin red S staining, and occurred exclusively in osteogenic medium (Figure 1B and 1D), indicating that PBMC-derived signals synergize with, but do not replace, osteogenic signals generated by the differentiation medium. These data show that PBMCs can promote MSC to OB differentiation. To identify the population within the PBMCs responsible for this osteogenic effect, T cells, B cells and monocytes were systematically depleted from PBMCs and the depleted populations co-cultured with MSCs at the ratio of 10∶1. Depletion of monocytes led to a 5-fold decrease in PBMC-mediated ALP induction in MSCs (Figure 1E), indicating that monocytes are responsible for the elevated MSC-ALP activity. The contribution of monocytes to increasing MSC-ALP activity was further verified using enriched T cell, B cell and monocyte populations in MSC co-cultures. The addition of enriched T cell and B cell populations did not have any effect on basal MSC ALP activity of mono-culture controls, thus indicating that neither T cells nor B cells could promote or inhibit MSC differentiation in their resting state (Figure 1F). However, in the presence of monocyte-enriched populations there was a significant increase in MSC-ALP activity (3-fold as compared to control) (Figure 1F). In the presence of monocyte-enriched populations, increased bone nodule formation was also observed (Figure 1G and 1H). To determine whether differentiated myeloid cells also support osteogenesis, monocytes were differentiated to macrophages for 5 days using either M-CSF or GM-CSF prior to co-culture with MSCs. As shown in Figure 1I, both cell types induced MSC differentiation, indicating that cells of the monocytic lineage are capable of producing osteo-inductive factors.

### Monocyte-derived Soluble Factors Promote OB Differentiation and Signaling in MSCs

**Figure 2:**
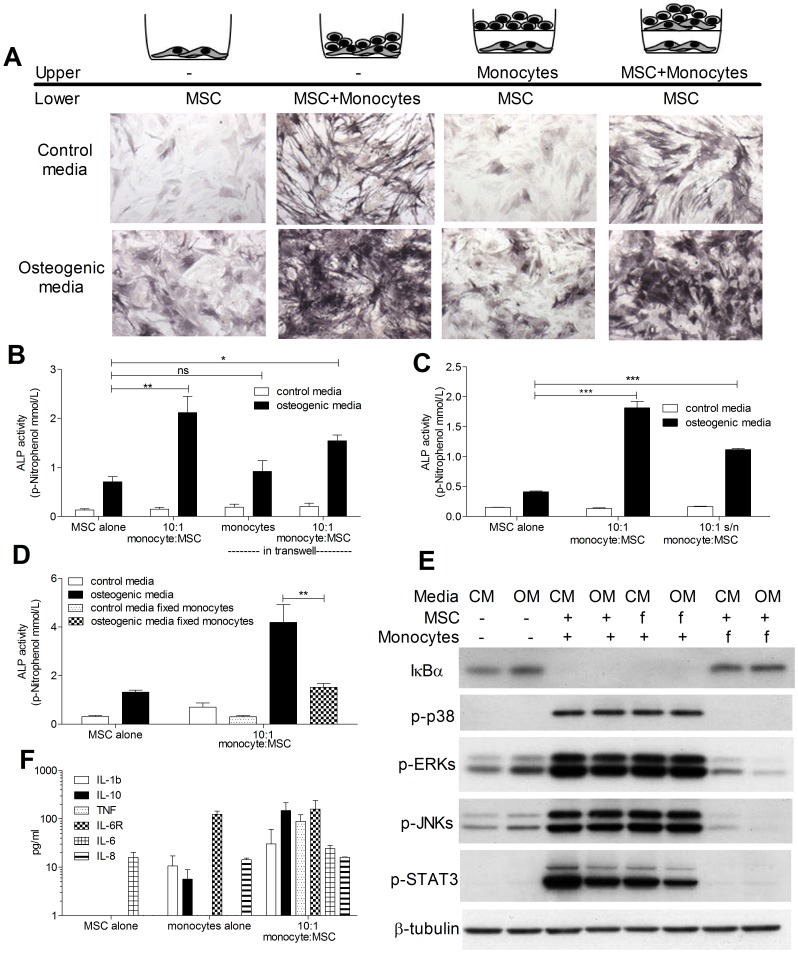
Osteogenic induction requires cell contact and monocyte-derived soluble factors. MSCs were cultured either alone, with 10∶1 monocytes in contact, with monocytes separated by a 0.4 µm pore size cell culture insert (upper chamber) or with a co-culture of 10∶1 monocyte:MSC in the cell culture insert (upper chamber), in the presence or absence of osteogenic stimuli. After 7 days, MSC ALP was assessed by staining (A) and quantification of enzyme activity (B). ALP activity induced by 10∶1 monocyte:MSC co-cultures was compared to that induced by treatment with supernatant from co-cultures incubated for 24 hours (10∶1 s/n) (C). ALP activity of MSCs was also measured after 7 days of co-culture with either fresh of fixed monocytes (fixed in 0.05% glutaraldehyde for 1 min followed by 1 min in 0.2 M glycine) (D). MSC were treated for 30 mins with supernatant from monocyte:MSC co-cultures or monocyte:MSC co-cultures where either monocytes or MSCs had been fixed (f), in control media (CM) or in osteogenic media (OM) and then lysed (E). MSCs alone in CM and OM were used as controls. MSC lysates (10 µg per condition) were subjected to WB for detection of IκBα, p-p38, pERKs, p-JNKs and pSTAT3. Anti-β-Tubulin was used as a loading control. The levels of IL-1β, IL-10, TNFα, IL-6, IL6R and IL-8 were measured in culture media from MSC or monocyte alone cultures or 10∶1 monocyte:MSC co-cultures (F). Phase contrast pictures (10X) are representative of three independent experiments performed. Graphs show means ± SEM of at least three independent experiments performed in triplicate. *p≤0.05, **p≤0.01, ***p≤0.001 Blots are representative of three independent experiments performed.

The ability of monocytes to promote osteogenic differentiation of MSCs was found to require cell contact because the separation of the two cell types using 0.4 µM pore size semi-permeable transwells significantly abrogated the increase in ALP staining and activity seen in the co-cultures in contact (Figure 2A and B). However, co-culturing MSCs and monocytes in the upper chambers of the transwells was sufficient to induce an increase in ALP staining and activity of MSCs cultured alone in the lower chamber of the transwells, indicating that cell contact leads to the production of soluble mediator(s) that are able to induce osteogenesis (Figure 2A and B). Indeed, conditioned media obtained from 10∶1 monocyte/MSC overnight co-cultures increased ALP activity when added to MSCs alone (Figure 2C). Soluble factors responsible for MSC osteogenesis were monocyte-derived as fixing monocytes prior to co-culture with MSCs abrogated ALP induction (Figure 2D). The addition of co-culture supernatant to MSCs alone cultures showed that monocyte-derived factors led to the activation of various signaling pathways as shown by IκBα degradation, increased phosphorylation of the MAPKs; p-38, ERK and JNK, and STAT3 within 30 minutes (Figure 2E). Supernatants from co-cultures of MSCs and fixed monocytes that failed to induce MSC differentiation (Figure 2D) also failed to activate any of these signaling pathways (Figure 2E). Conversely, fixing the MSCs prior to co-culture with monocytes did not inhibit signaling in response to the supernatants showing that contact with the cell surface of MSCs is sufficient to induce the production of monocyte-derived factors necessary for cell signaling in the MSCs (Figure 2E). ELISA of the co-culture supernatants identified elevated cytokines in the media including IL-1β, IL-10, TNFα, IL-6 and IL-8 (Figure 2F). Of these cytokines only IL-1β could increase ALP activity when added directly to MSC cultures in increasing concentrations (Figure S1A). However, addition of IL-1β neutralising antibody failed to abrogate the monocyte-induced ALP activity in MSC indicating that it is not the mediator of monocytes’ osteogenic activity (Figure S1B). Addition of IL-10, IL-6 and IL-8 (Figure S1D, E, G) had no direct effect on MSC-ALP activity whilst TNFα was inhibitory at high concentrations (>10 ng/ml) (Figure S1C). In addition to a lack of effect on ALP activity, IL-10 and IL-6 did not cause STAT3 phosphorylation in MSCs in contrast to the 10∶1 co-culture supernatant (Figure S1F, 1H). Thus, despite being well known activators of the STAT3 signalling pathway, IL-6 and IL-10 were not responsible for the STAT3 activation observed in monocyte/MSC co-cultures.

Likewise, BMP2, BMP4 and BMP7 and TGFβ1 levels were not elevated in co-culture conditioned media (Table S1). Furthermore, neutralisation of BMP2, 3, 4, 7 and TGFβ1 in these co-cultures did not inhibit the ability of macrophages to induce MSC ALP activity indicating that these factors are not mediators of the monocyte/macrophage-mediated osteogenic induction (Figure S2).

### STAT3 Activation Promotes Osteoblast Differentiation

**Figure 3:**
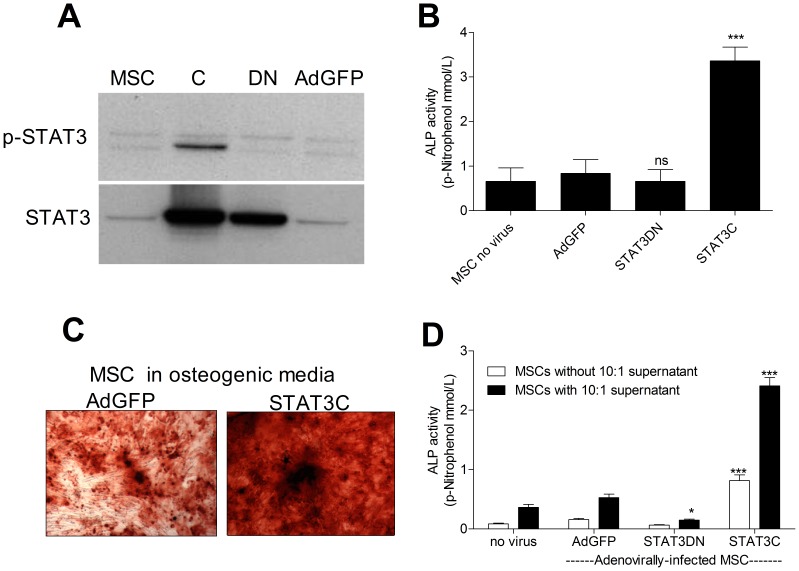
OSM mediates monocyte osteogenic effect through STAT3 signaling. The levels of pSTAT3 and STAT3 were assessed using WB in MSCs infected either with STAT3C (50 M.O.I.) or STAT3DN (100 M.O.I.) adenoviruses in osteogenic media. GFP adenoviral infection was used as a control (100 M.O.I.) (A). MSC ALP activity was measured 7 days after infection either with STAT3DN, STAT3C or AdGFP (B). MSC infected with the STAT3C or AdGFP viruses were also kept for 21 days in osteogenic media when bone nodule formation was assessed using Alizarin Red S staining (C). MSC infected with either the STAT3DN, STAT3C or AdGFP were kept with or without conditioned supernatant from separate monocyte:MSC co-cultures (10∶1 s/n) for 7 days when ALP activity was quantified (D). Blots are representative of three independent experiments performed. Graphs show means ± SEM of three independent experiments performed in triplicate. Phase contrast pictures (10X) are representative of three independent experiments performed. *p≤0.05, ***p≤0.001.

Investigations into the signaling pathways regulating OB differentiation and function have tended to focus on factors that are downstream of BMP, Wnt, Notch and PTH/PTHrP [12], [13], [14]. Far less has been reported on the NFκB, MAPK and JAK/STAT pathways. It has been postulated that NFκB signaling may be detrimental to OB differentiation [41] whilst the MAPKs, particularly ERK, have been reported to act upstream of Runx2 and Osterix to increase their mRNA and protein levels but also to enhance their transcriptional activity [42], [43], [44]. In view of the strong regulation of STAT3 in our co-cultures, we assessed the requirement for STAT3 signaling in MSC differentiation. Adenoviruses expressing a constitutively active (STAT3C) or a dominant negative (STAT3DN) form of STAT3 were used to infect MSCs (Figure 3A). STAT3C led to a super-induction in ALP activity levels compared to control adenoviral vector (AdGFP) (Figure 3B) as well as increased formation of mineralised bone nodules (Figure 3C), whilst little effect was seen in STAT3DN MSCs. To validate whether the activation of STAT3 in MSCs was required for osteogenesis induced by monocytes, STAT3C and STAT3DN infected MSCs were cultured in monocyte/MSC co-culture supernatants. STAT3DN suppressed ALP induction in MSC cultured with co-culture supernatants (Figure 3D) indicating that the activation of STAT3 in MSCs is required for the osteogenic effect of the co-culture supernatants. The combination of STAT3 constitutive activation in MSCs and treatment with 10∶1 co-culture supernatant caused a super induction of ALP activity (Figure 3D), further indicating that co-culture supernatants contain other factors that can act in concert with STAT3 for OB differentiation.

### Microarray Analysis of STAT3 Activated Factors in MSCs

To elucidate the mechanisms behind the regulation of osteogenesis by STAT3, genome-wide changes in gene expression of MSCs infected with the STAT3 viruses or the control adenoviral vector AdGFP were assessed using Illumina Human HT-12 v3.0 Expression BeadChip microarrays. Prior to the microarray analysis, it was verified by real time RT-PCR that 24 hours after infection with STAT3C was sufficient to cause a two-fold up-regulation of ALP mRNA in MSCs (data not shown). MSCs from five different donors were infected with the adenoviral constructs and cultured for 24 hours after which RNA was isolated. Raw data normalization, filtering and analysis were performed using GeneSpring 11.5. Genes with significantly altered expressions between STAT3C or STAT3DN and AdGFP were identified by one way ANOVA with Benjamini-Hochberg multiple testing correction, with p<0.01. The entities satisfying the significance analysis were subsequently used for the fold change analysis that is calculated by dividing the average normalised intensity for a given gene in treated samples from the respective control values. We identified 479 genes that were differentially regulated between STAT3C MSC and AdGFP MSC by at least 1.5-fold, of which 314 were up-regulated and 165 down-regulated. The top 50 genes of both groups according to highest fold changes are listed in Tables 1 and 2, respectively. In contrast, only five genes were differentially expressed between STAT3DN MSC and AdGFP MSC by at least 1.5-fold. The genes differentially regulated by STAT3C included those previously reported to be STAT3 targets such as SOCS3 [45], lipopolysaccharide-binding protein (LBP) [46], Jun, JunB [47], Myc [48], HIF1A [49], Pim1 [50] and VEGF [51]. Furthermore, a significant number of genes found to be up-regulated (including LBP, IL1R1, SERPIN, IGFBP1, CCL2, EFNA1, ANGPTL4, BCL3) or down-regulated (including IFIT1, MX1, OAS1, MYC, PDK4) by STAT3C were consistent with those identified to be regulated by STAT3 in lung epithelial cells [52], indicating that these genes are not unique to OB differentiation. In contrast, up-regulation of OSMR and PDGFRA, also induced by STAT3C in mouse embryonic fibroblasts [53], may be more relevant to OB differentiation of MSC.

**Table 1:** Genes up-regulated by STATC.

Symbol	Definition	Fold change STAT3C v AdGFP
**LBP**	Lipopolysaccharide binding protein	10.31
**IL1RL1**	Interleukin 1 receptor-like 1	9.95
**SAA2**	Serum amyloid A2	8.37
**CCL8**	Chemokine (C-C motif) ligand 8	7.98
**NFE2**	Nuclear factor (erythroid-derived 2)	6.67
**SERPINB4**	Serpin peptidase inhibitor clade B (ovalbumin), member 4	5.99
**PMAIP1**	Phorbol-12-myristate-13-acetate-induced protein 1	5.67
**F3**	Coagulation factor III (thromboplastin, tissue factor)	5.18
**EFNA1**	Ephrin A1	4.96
**IL6**	Interleukin 6 (interferon, beta 2)	4.92
**CHI3L2**	Chitinase 3-like 2	4.55
**SOCS3**	Suppressor of cytokine signaling 3	4.55
**SAA1**	Serum amyloid A1	4.43
**FLJ22447**	PREDICTED: hypothetical gene supported by AK026100	4.42
**WWC1**	WW and C2 domain containing 1	4.33
**SERPINB3**	Serpin peptidase inhibitor clade B (ovalbumin), member 3	4.27
**NAMPT**	Nicotinamide phosphoribosyltransferase	4.25
**MT1M**	Metallothionein 1 M	4.22
**CH25H**	Cholesterol 25-hydroxylase	4.08
**PIM1**	pim-1 oncogene	4.07
**STEAP4**	STEAP family member 4	3.76
**KIAA1199**	KIAA1199	3.68
**CA2**	Carbonic anhydrase II	3.65
**TNC**	Tenascin C	3.58
**ANGPTL4**	Angiopoietin-like 4	3.39
**RCAN1**	Regulator of calcineurin 1	3.39
**ACHE**	Acetylcholinesterase	3.38
**HP**	Haptoglobin	3.33
**GSDMC**	Gasdermin C	3.27
**IL1R2**	Interleukin 1 receptor, type II	3.22
**BCL3**	B-cell CLL/lymphoma 3	3.18
**IL1R1**	Interleukin 1 receptor, type I	3.03
**RGS16**	Regulator of G-protein signaling 16	3
**MAFB**	v-maf musculoaponeurotic fibrosarcoma oncogene homolog B (avian)	2.99
**TDO2**	Tryptophan 2,3-dioxygenase (TDO2)	2.93
**KRTAP1–5**	Keratin associated protein 1–5	2.92
**SLC2A3**	Solute carrier family 2 (facilitated glucose transporter), member 3	2.92
**PLAU**	Plasminogen activator, urokinase	2.90
**C10orf10**	Chromosome 10 open reading frame 10	2.90
**IGF1**	Insulin-like growth factor 1 (somatomedin C)	2.87
**GLDN**	Gliomedin	2.83
**G0S2**	G0/G1switch 2	2.83
**TNFRSF21**	Tumor necrosis factor receptor superfamily, member 21	2.82
**SERPINA3**	Serpin peptidase inhibitor, clade A (alpha-1 antiproteinase, antitrypsin), member 3	2.78
**CXCL5**	Chemokine (C-X-C motif) ligand 5	2.76
**MT1X**	Metallothionein 1X	2.74
**STEAP1**	PREDICTED: Six transmembrane epithelial antigen of the prostate 1	2.74
**CCL7**	Chemokine (C-C motif) ligand 7 (CCL7)	2.69
**INSIG1**	Insulin induced gene 1	2.68
**NOD1**	Nucleotide-binding oligomerization domain containing 1	2.64

The table lists the 50 most highly significantly up-regulated transcripts in MSC STAT3C compared to MSC AdGFP (with p<0.01 cutoff using one way ANOVA with Benjamini-Hochberg multiple testing correction). Values shown are the medians from the five donors.

**Table 2:** Genes down-regulated by STATC.

Symbol	Definition	Fold change STAT3Cv AdGFP
**KRTAP1–5**	Keratin associated protein 1–5	−2.92
**IFIT1**	Interferon-induced protein with tetratricopeptide repeats 1	−2.68
**C10orf54**	Chromosome 10 open reading frame 54	−2.68
**LOC728956**	PREDICTED: Similar to keratin associated protein 1.5	−2.63
**LOC728946**	PREDICTED: Similar to keratin associated protein 1-1	−2.49
**FAM43A**	Family with sequence similarity 43, member A	−2.41
**PGBD3**	PiggyBac transposable element derived 3	−2.37
**LOC728951**	PREDICTED: Similar to keratin associated protein 1–3	−2.32
**MARCH4**	Membrane-associated ring finger (C3HC4) 4	−2.29
**DKK1**	Dickkopf homolog 1 (Xenopus laevis)	−2.28
**ADH1A**	Alcohol dehydrogenase 1A (class I), alpha polypeptide	−2.28
**OSAP**	Ovary-specific acidic protein	−2.18
**RGS4**	Regulator of G-protein signalling 4	−2.17
**CTNNB1**	Catenin (cadherin-associated protein), beta 1, 88 kDa	−2.13
**DUSP10**	Dual specificity phosphatase 10	−2.13
**ADRA1B**	Adrenergic, alpha-1B-, receptor	−2.13
**PPP1R3C**	Protein phosphatase 1, regulatory (inhibitor) subunit 3C	−2.12
**SORBS2**	Sorbin and SH3 domain containing 2	−2.12
**C4orf49**	Chromosome 4 open reading frame 49	−2.11
**PDK4**	Pyruvate dehydrogenase kinase, isozyme 4	−2.10
**TNFRSF19**	Tumor necrosis factor receptor superfamily, member 19	−2.08
**SLC40A1**	Solute carrier family 40 (iron-regulated transporter), member 1	−2.05
**CDC42EP3**	CDC42 effector protein (Rho GTPase binding) 3	−2.05
**CPA4**	Carboxypeptidase A4	−2.04
**LRRN3**	Leucine rich repeat neuronal 3, transcript variant 1	−2.01
**CLIC3**	Chloride intracellular channel 3	−1.97
**ARPC2**	Actin related protein 2/3 complex, subunit 2, 34 kDa	−1.97
**ANGPT1**	Angiopoietin 1	−1.93
**NR2F1**	Nuclear receptor subfamily 2, group F, member 1	−1.93
**EDN1**	Endothelin 1	−1.92
**IRX5**	Iroquois homeobox protein 5	−1.92
**CITED2**	Cbp/p300-interacting transactivator, with Glu/Asp-rich carboxy-terminal domain 2	−1.92
**LYPD6B**	LY6/PLAUR domain containing 6 B	−1.91
**UNQ1940**	HWKM1940	−1.90
	K-EST0187371 L5HLK1 Homo sapiens cDNA clone L5HLK1-32-B12 5	−1.89
**MEOX2**	Mesenchyme homeobox 2	−1.89
**STC2**	Stanniocalcin 2	−1.88
**TSC22D3**	TSC22 domain family, member 3	−1.87
**PIK3IP1**	Phosphoinositide-3-kinase interacting protein 1	−1.87
**EBF3**	Early B-cell factor 3	−1.86
**FHL1**	Four and a half LIM domains 1	−1.86
**EFHD1**	EF-hand domain family, member D1	−1.85
**DHRS3**	Dehydrogenase/reductase (SDR family) member 3	−1.84
**ERCC6**	Excision repair cross-complementing rodent repair deficiency, complementation group 6	−1.81
**TXNRD1**	Thioredoxin reductase 1	−1.80
**TIAM2**	T-cell lymphoma invasion and metastasis 2	−1.79
**KCTD12**	Potassium channel tetramerisation domain containing 12	−1.78
**TMEM47**	Transmembrane protein 47	−1.78
**IFIT2**	Interferon-induced protein with tetratricopeptide repeats 2	−1.77
**ADH1B**	Alcohol dehydrogenase IB (class I), beta polypeptide	−1.76

The table lists the 50 most highly significantly down-regulated transcripts in MSC STAT3C compared to MSC AdGFP (with p<0.01 cutoff using one way ANOVA with Benjamini-Hochberg multiple testing correction). Values shown are the medians from the five donors.

The genes differentially regulated in STAT3C MSC v AdGFP MSC were classified ontologically using PANTHER classification system pathways which also statistically determines over- or under- representation of genes in a particular pathway (Table 3). Pathway analysis showed significant over-representation of genes related to inflammation mediated by chemokine and cytokine signaling pathway including many chemokines such as CCL2, CCL3L, CCL8 and CCL7 (Table 4), which are known to exhibit chemoattractant activity for monocytes and macrophages. Other significantly represented pathways were angiogenesis and interleukin signalling pathways, TGF-β signalling pathway, cytoskeletal regulation by Rho GTPase and Toll receptor signalling pathway. In addition, each gene was researched against published literature using PubMed to identify relations to OB differentiation and function (Table 4). There was no significant increase in the expression of any of the BMP or TGFβ family members. There was a significant increase in ALP as expected, as well as in the OB transcription factor, Runx2, accompanied by a decrease in the Wnt signalling inhibitor, Dkk1.

**Table 3:** Pathways identified by ontological analysis to be over-represented in MSC STAT3C versus MSC AdGFP.

Pathways	Homo sapiens genes	Regulated genes 1.5 fold STAT3C v AdGFP	Expected	P value
**Inflammation mediated by chemokine and** **cytokine signaling pathway**	283	23	6.21	1.33E−07
**Angiogenesis**	191	12	4.19	1.27E−03
**Unclassified**	17337	362	380.51	6.54E−03
**Plasminogen activating cascade**	18	3	0.4	7.63E−03
**Insulin/IGF pathway-mitogen activated protein** **kinase kinase/MAP kinase cascade**	35	4	0.77	7.84E−03
**Interleukin signaling pathway**	161	9	3.53	1.01E−02
**Oxidative stress response**	60	5	1.32	1.11E−02
**Alzheimer disease-presenilin pathway**	122	7	2.68	1.94E−02
**Blood coagulation**	48	4	1.05	2.23E−02
**Cytoskeletal regulation by Rho GTPase**	98	6	2.15	2.23E−02
**Interferon-gamma signaling pathway**	29	3	0.64	2.68E−02
**Heterotrimeric G-protein signaling pathway-Gq** **alpha and Go alpha mediated pathway**	134	7	2.94	3.01E−02
**Ras Pathway**	79	5	1.73	3.15E−02
**TGF-beta signaling pathway**	145	7	3.18	4.29E−02
**Threonine biosynthesis**	2	1	0.04	4.29E−02
**Lysine biosynthesis**	2	1	0.04	4.29E−02
**Carnitine metabolism**	2	1	0.04	4.29E−02
**Carnitine and CoA metabolism**	2	1	0.04	4.29E−02
**FAS signaling pathway**	36	3	0.79	4.59E−02
**Toll receptor signaling pathway**	62	4	1.36	4.91E−02

Pathways identified by ontological analysis to be significantly overrepresented in the differentially regulated genes of the STAT3C MSCs versus AdGFP MSCs analysis.

**Table 4:** Selected genes associated with OB differentiation and/or function.

Symbol	Definition	Fold change STAT3Cv AdGFP	References
**SAA2**	Serum amyloid A2	8.37	[69]
**EFNA1**	Ephrin A1	4.97	[72], [87]
**IL6**	Interleukin 6	4.92	[88]
**SAA1**	Serum amyloid A1	4.43	[69]
**MT1M**	Metallothionein 1 M	4.22	[89]
**TNC**	Tenascin C	3.58	[90]
**RCAN1**	Regulator of calcineurin 1	3.39	[91]
**IL1R2**	Interleukin 1 receptor, type II	3.23	[92]
**IL1R1**	Interleukin 1 receptor, type I	3.04	[92]
**RGS16**	Regulator of G-protein signalling 16	3.00	[88]
**PLAU**	Plasminogen activator, urokinase	2.90	[93]
**IGF1**	Insulin-like growth factor 1	2.87	[68], [94]
**CXCL5**	Chemokine (C-X-C motif) ligand 5	2.76	[95]
**MT1X**	Metallothionein 1X	2.73	[89]
**CCL7**	Chemokine (C-C motif) ligand 7	2.67	[88]
**ALPL**	Alkaline phosphatase, liver/bone/kidney	2.60	[96]
**RGS2**	Regulator of G-protein signalling 2	2.57	[88]
**DKK1**	Dickkopf homolog 1 (Xenopus laevis)	−2.28	[97]
**IGFBP1**	Insulin-like growth factor binding protein 1	2.27	[98]
**MT1H**	Metallothionein 1 H	2.24	[89]
**RGS4**	Regulator of G-protein signalling 4	−2.17	[88]
**OSMR**	Oncostatin M receptor	2.05	[54]
**CCL2**	Chemokine (C-C motif) ligand 2	2.03	[95]
**RUNX2**	Runt-related transcription factor 2	1.95	[99]
**MTE**	Metallothionein E	1.76	[89]
**PDGFRA**	Platelet-derived growth factor receptor-alpha	1.76	[100]
**MT1E**	Metallothionein 1 E	1.67	[89]
**MT1G**	Metallothionein 1 G	1.67	[89]
**LIFR**	Leukemia inhibitory factor receptor alpha	1.66	[54]
**IL24**	Interleukin 24	1.50	[88]

Differentially regulated transcripts in MSC STAT3C compared to MSC AdGFP associated with osteogenic differentiation and function.

### Oncostatin M Mediates the abIlity of Monocytes to Increase MSC-OB Differentiation

**Figure 4:**
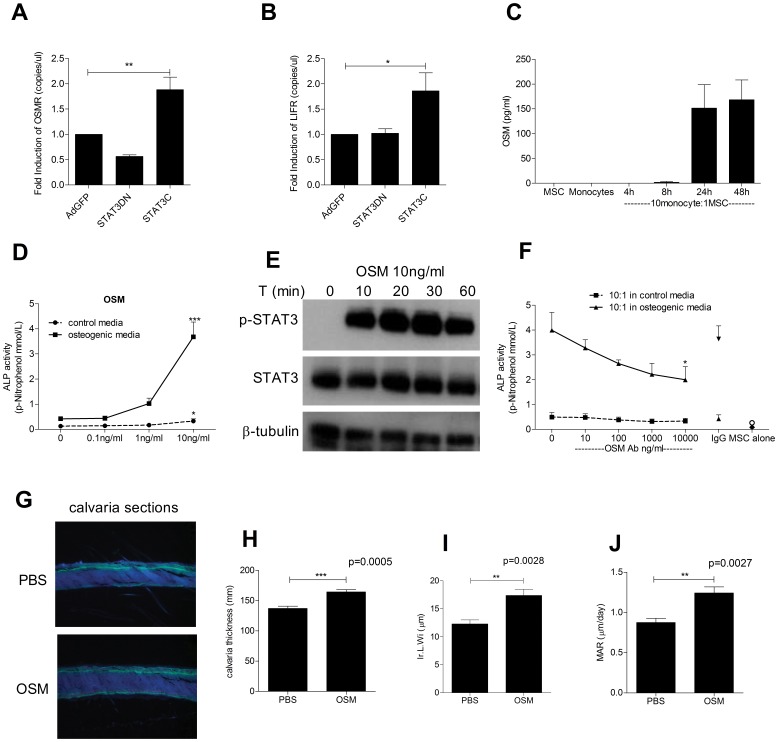
OSM mediates monocyte osteogenic effect. RNA was isolated from STAT3C, STAT3DN and AdGFP infected MSC after 24 h of infection and the levels of OSMR (A) and LIFR (B) mRNA were measured using real-time RT-PCR. Graphs are expressed as fold induction compared to AdGFP infected MSC. OSM levels were assessed in supernatants from either MSCs or monocytes cultured alone or MSC/monocytes co-cultured for 4 h, 8 h, 24 h and 48 h using OSM ELISA (C). MSC (1×10^4^) were treated with increasing concentration of human recombinant OSM in either control or osteogenic media and ALP activity was assessed after 7 days (D). MSCs were treated with 10 ng/ml of OSM for increasing time (10, 20, 30 and 60 mins), cultures were lysed and 10 µg of protein per condition was subjected to WB for detection of pSTAT3, STAT3 and β-tubulin (E). OSM neutralising antibody was added to MSC:monocyte co-cultures at increasing concentration (10, 100, 1000 and 10000 ng/ml) in control or osteogenic media. Isotype control was also added to match the highest concentration. ALP activity was quantified after 7 days (F). Blots are representative of three independent experiments performed. Graphs show means ± SEM of at least three independent experiments performed in triplicate. *p≤0.05 ***p≤0.001. Calcein labeled calvaria sections from mice injected daily with either recombinant murine OSM or PBS observed under fluorescent light (20X) (G). Local administration of 0.2 µg/d mOSM for 5 days in C57BL/6 mice significantly increased calvarial thickness (H), inter-label width (Ir.L.W.) (I) and mineral apposition rate (MAR) (J). Data are shown as mean + SEM, 6 mice/group.

Overexpression of STAT3C also led to the up-regulation of OSMR (Figure 4A) and LIFR (Figure 4B) mRNA as assessed by RT-PCR; OSM has been reported to act via both receptors to enhance OB activity [54]. Due to the up-regulation of these receptors, the levels of OSM produced in the monocyte/MSC co-cultures were examined. Although OSM was not produced by MSCs or monocytes cultured alone, its levels increased over the course of 48 hours in monocyte/MSC co-cultures (Figure 4C). When added to MSC cultures directly, recombinant human OSM induced osteogenic differentiation in a dose dependent manner as assayed by quantification of ALP activity (Figure 4D). In addition, MSCs cultured in the presence of OSM showed phosphorylation of STAT3 within 10 minutes as observed by western blot (Figure 4E). Conversely, the addition of OSM neutralizing antibodies in MSC/monocyte co-cultures abrogated the ALP induction in a dose dependent manner verifying OSM as a monocyte-derived factor with osteogenic activity (Figure 4F). The ability of OSM to stimulate bone formation *in vivo* was assessed by injecting mOSM over calvariae of 5-week-old male C57BL/6 mice. Calvarial thickness, inter-label width (Ir.L.W.) and mineral apposition rate (M.A.R.) were all significantly increased by mOSM treatment compared to PBS (Figure 4G–J). Thus, OSM produced after monocyte/MSC interactions is responsible for increased osteogenesis through the activation of STAT3 signaling which in addition acts as positive feedback to enhance the effects of OSM on MSCs by up-regulating the OSM and LIF receptors.

## Discussion

Previously there has been limited evidence regarding the direct regulation of OB differentiation by immune cells. Earlier studies suggested a possible role for activated T cells in influencing OB differentiation through soluble factors [55], [56], and haematopoietic stem cells have also been shown to regulate OB differentiation through the production of BMP2 and BMP6 [57]. More recently, resident macrophage populations found in bone have been demonstrated to serve as critical regulators of bone homeostasis [25] as well as repair in a murine tibia injury model [26]. However, the mechanisms and mediators of these important interactions remain elusive. These studies proposed that the close interactions between OBs and macrophages *in vivo* are critical for the integrity of bone remodelling sites as well as efficient mineralization. In this study, we show that monocytes and macrophages directly regulate osteogenic differentiation of MSCs through a mechanism that involves cell contact, leading to the production of OSM by monocytes and STAT3 signalling in the MSCs.

In support of our findings, Guihard and colleagues have recently shown that monocyte-macrophage production of OSM upon toll-like receptor (TLR) activation by liposaccharide or endogenous ligands such as poly I:C, can promote osteogenesis [58]. However, by specifically focusing on the responses in the context of activated monocytes and macrophages, their findings are only relevant to pathological settings such as bone inflammation, infection or injury. We find that it is the contact between monocytes and MSC that regulates the production of OSM by the monocytes and the up-regulation of the OSM and LIF receptors on the MSC. The use of non-activated monocytes and macrophages in our system makes our findings more relevant to the regulatory networks governing physiological bone formation and provide further evidence of the crucial role of monocyte/macrophages and their derived factors in these networks.

A number of cytokines including IL-1β, IL-10, IL-6 and TNFα were increased following contact between MSC and monocytes in agreement with previous studies [35], [37], [38], [59], [60], however these were not responsible for the enhanced osteogenesis induced by monocytes. Although some studies have identified a role for IL-6 in directly promoting osteogenic differentiation [61], [62], these were conducted on osteoblastic cell lines such as MG-63 or calvarial OBs. Additionally, the induction of STAT3 in co-cultures of MSCs with macrophages has also been previously reported suggesting that this could be a mechanism by which MSCs promote an anti-inflammatory phenotype in macrophages [63]. However, as we show here, the activation of STAT3 signaling in the MSCs in co-cultures cannot be attributable to the up-regulation of IL-10 or IL-6 since both cytokines failed to induce STAT3 phosphorylation in MSCs.

Signalling through the JAK/STAT pathway has previously been shown to be important for OB differentiation. OB-specific disruption of the Stat3 gene via the Cre-LoxP recombination system using α1(I)-collagen promoter Cre transgenic mice results in a reduced rate of bone formation, leading to an osteoporotic phenotype while glycoprotein 130 (gp130) knock-in mice, in which STAT3 activation is enhanced, exhibit an osteosclerotic phenotype [64]. Thus STAT3 signaling in OBs plays a pivotal role in bone formation *in vivo*. In our system, the role of STAT3 signaling in mediating the osteogenic effects of monocytes was validated by the enhanced osteogenesis observed in MSCs overexpressing STAT3C and by the ability of a STAT3 DN virus to suppress MSC ALP activity induced by the osteogenic 10∶1 co-culture supernatants (Figure 3). These findings confirm that monocyte/macrophage-induced MSC osteogenesis occurs via the STAT3 signaling pathway and are consistent with a recent study which showed that the siRNA knockdown of STAT3 resulted in a significant reduction of MSC ALP activity following treatment with dexamethasone and BMP-2 [65]. By contrast, however, in a separate study eradication of STAT3 signalling also using siRNA has been found to result in highly efficient osteogenic differentiation induced by BMP-2 albeit also showing that BMP2/4 induced STAT3 tyrosine phosphorylation [66]. In spite of these contradictory findings, the majority of published literature as well as our investigations stipulate an important role for STAT3 signaling positively influencing MSC osteogenic differentiation.

Importantly a great number of genes known to be associated with OB differentiation were identified to be induced by STAT3C in our microarray screening. As expected, ALP was shown to be up-regulated by 2.6 fold in MSC STAT3C compared to MSC AdGFP. Furthermore, the bone-specific transcription factor Runx2 expression was up-regulated by 1.95 fold in STAT3C MSC. In contrast, expression of Dkk1, a negative regulator of Wnt signaling and osteogenic differentiation [18], [67], was down-regulated by 2.28 fold in STAT3C MSC. These observations suggest that the overall balance is strongly in favour of OB differentiation. Furthermore, the rapid increase in both Runx2 and ALP suggests that these two genes are direct targets of STAT3 although further studies are required to test this hypothesis. Interestingly, both IGF1, which is known to promote osteogenic differentiation [68] as well as mediate other anabolic effects, and IGF binding protein 1 (IGFBP1) were also found to be up-regulated. Serum amyloid A2 and A1 were up-regulated in the STAT3C MSC yet SAA2 was down-regulated 1.56 fold in the STAT3DN MSC. Serum amyloid A (SAA) proteins are a family of cytokine-induced acute-phase proteins that participate in the acute inflammatory process through the recruitment of immune cells and the induction of enzymes that degrade extracellular matrix. Expression of SAA1/2 has been demonstrated in non-differentiated MSCs and was shown to increase during osteogenic differentiation [69]. EphrinA1, the ligand receptor for EphA2 a membrane bound receptor tyrosine kinase [70], was up-regulated by STAT3C. Recent studies suggest that Eph/eprhin molecules regulate bone homeostasis mediating the crosstalk between OBs and OCs. More specifically, bidirectional signaling through ephrin A2-EphA2 was shown to enhance osteoclastogenesis while suppressing osteoblastogenesis [71], while recently ephrin B molecules were shown to be required for mineralisation and glycosaminoglycan synthesis in *in vitro* MSC cultures [72]. These findings may suggest a role for ephrinA1 in regulating osteogenesis.

STAT3C induced the expression of OSMR and LIFR (by 2 fold and 1.66 fold respectively) which together with gp130 form receptor complexes for OSM. OSM belongs to the IL-6 family of cytokines which signal primarily through JAK/STAT3 but also MAPKs pathways. OSM signaling through gp130 and STAT3 has been linked to OB differentiation [73]. Receptor components gp130, LIFR and OSMR that bind OSM are expressed by bone marrow osteoblastic cells, OB cell lines as well as mouse calvaria OBs, bone lining cells and osteocytes [54], [74]. In support of our findings, OSM can promote osteogenic differentiation and mineralisation both *in vitro* and *in vivo* whilst IL-6 and LIF did not induce mineralisation [54], [75], [76], [77]. Transgenic mice overexpressing OSM develop osteopetrotic bones and enlarged hind limbs, likely by stimulation of bone formation and concomitant inhibition of bone resorption [78]. In addition, monocytes and macrophages are known producers of OSM [79] and it has been shown that PGE_2_ can induce OSM expression in microglia, the resident macrophages of the brain, as well as in monocytes and macrophages of mouse and human origin [80]. Similar to the induction of STAT3 signaling in MSCs shown here, OSM can activate STAT3 in OBs [81]. Stimulation of ALP activity by OSM was abrogated by tyrosine kinase inhibitors as well as a threonine/serine kinase inhibitor, but only minimally affected by a specific inhibitor of MAPK phosphorylation, indicating a primary role for the JAK/STAT pathways mediating OSM osteogenic properties [61]. OSM signalling through STAT3 has also been shown to directly target Wnt5a [82], [83] which promotes osteogenic differentiation of MSCs.

Following the identification of OSM as an important mediator of OB regulation by monocytes and macrophages, it would be pertinent to investigate the expression of OSM in the distinct macrophage population, termed Osteomacs, found to be intercalated throughout bone tissues and specifically located adjacent to the layer of bone lining cells [25]. Indeed, Walker and colleagues have recently described that OSM is expressed throughout murine bone tissue where it regulates OBs acting through both the OSMR and LIFR with distinct outcomes [54]. More specifically, signaling through the OSMR was associated with early stromal cell lineage commitment towards OBs through up-regulation of C/EBPβ and C/EBPδ whereas OSM action through LIFR was associated with inhibition of sclerostin expression in osteocytes.

In conclusion, we show that monocytes/macrophages can potently induce the osteogenic differentiation of human MSCs in a mechanism that involves the production of OSM by the former that activates STAT3 in the latter. The phosphorylation of STAT3 in the MSCs up-regulates RUNX2 and ALP expression, along with other OB-associated genes, and down-regulates Dkk1 leading to the differentiation of MSC into OB. Additionally, STAT3 activation in the MSCs leads to the up-regulation of OSMR and LIFR, thereby amplifying the effects of OSM.

## Methods

### Cell Culture and Reagents

Adult human MSCs were commercially purchased (Product No PT-2501, Lonza Group, Basel, Switzerland) or were isolated from bone marrow aspirates. This study has been approved by the National Research Ethics Service - Riverside Research Ethics Committee (REC Ref. No. 07/H0706/81) for the study of molecular mechanisms and pathways of chronic inflammatory and degenerative diseases. Human samples used in this study were obtained with written informed consent. Samples were collected at the time of musculoskeletal surgery for joint replacement or following traumatic injury.

MSCs were maintained in DMEM Glutamax media supplemented with 10% FBS (PAA, Austria) and 1% penicillin/streptomycin (PAA, Austria) and used between passage 3 and 8. Prior to their use in experiments MSCs were verified for their plastic-adherence, expression of cell surface markers CD105, CD73 and CD90 but lack of CD45, CD34 and HLA-DR molecules as well as tri-lineage differentiation to osteoblasts, adipocytes and chondrocytes in the presence of standard differentiation cocktails *in vitro*. Peripheral blood mononuclear cells (PBMC) were prepared from buffy coat fractions (North London Blood Service, Colindale, UK) by density gradient centrifugation. Depletion and enrichment of T cells, B cells and monocytes was performed using CD3, CD19 and CD14 Dynabeads and negative selection kits respectively (Dynal® T cell Negative Isolation Kit Ver II for T cells, Dynal® B Cell Negative Isolation Kit for B cells and Dynabeads® MyPure™ Monocyte Kit 2 for monocytes) all from Invitrogen, according to manufacturer’s instructions. Recombinant IL-1β, IL-6, IL-8, TNFα, IL-10 and OSM were purchased from Peprotech and all corresponding neutralising antibodies were from R&D systems. For real-time reverse transcription PCR Inventoried TaqMan® Gene expression Assays for OSMR (Hs00384278_m1) and LIFR (Hs01123581_m1) were used (Applied Biosystems) with Reverse Transcriptase qPCR™ Mastermix No ROX (Eurogentec). All other reagents were purchased from Sigma-Aldrich unless otherwise stated.

### MSC Differentiation

MSCs were cultured in osteogenic medium: D-MEM Glutamax supplemented with 100 nM dexamethasone, 50 µg/ml ascorbic acid 2-phosphate and 10 mM β-glycerophosphate. After 7 days, cells were either fixed with acetone:ethanol and stained for ALP using Sigma FAST™ BCIP/NBT tablet (Sigma-Aldrich) or their ALP activity was determined using LabAssay™ ALP kit (Wako, Japan). Bone nodule formation was assessed at 21 days by alizarin red S staining and quantification of dye content [84].

### Western Blot

Cell lysates were prepared in lysis buffer (25 mM HEPES (pH 7.0), 150 mM NaCl, and 1% Nonidet P-40), containing protease inhibitor cocktail (Roche Biochemicals, USA), and then electrophoresed on 10% SDS polyacrylamide gels, followed by electrotransfer of proteins onto PVDF transfer membranes (Perkin Elmer Life Sciences Inc., USA). Membranes were blocked in 8% non-fat dry milk/PBS (0.05%)/Tween and incubated overnight at 4°C with polyclonal primary antibodies against pSTAT3 (Tyr705), p-p38, pERKs, pJNKs (all from Cell Signaling Technology), STAT3, IκBα (both from Santa Cruz) or β-tubulin (Sigma-Aldrich). Horseradish peroxidase-conjugated anti-mouse IgG or anti-rabbit IgG (Amersham Biosciences) were used as secondary antibodies at a dilution of 1∶2000. Bound antibody was detected using the enhanced chemiluminescence kit (Amersham Biosciences) and visualized using Hyperfilm MP (Amersham Biosciences).

### ELISA

The levels of various cytokines in culture supernatants were measured using a Human Cytokine 30-plex Luminex assay kit (Invitrogen). OSM levels were measured using the Human OSM DuoSet from R&D systems.

### Adenoviral Infection

Recombinant adenoviruses expressing STAT3 constitutively active (C; A661C and N663C), STAT3 dominant negative (DN; Y705F) and green fluorescent protein (GFP) were used to infect MSC [85], [86]. Adenoviral infection efficiency was assessed by western blotting.

### Microarray

The microarray study was performed using the HumanHT-12 v3 expression BeadChips on the Illumina Whole-Genome Gene Expression analysis platform. MSCs from five different donors were infected with STAT3C, STAT3DN or AdGFP adenoviral constructs as described above and independent chips were used for each donor. Total RNA was extracted from each sample using the QIAamp RNA Blood Mini Kit (Qiagen Ltd, Crawley, West Sussex, UK) according to manufacturer’s instructions. Isolated RNA was quantified and RNA quality assessed using the RNA6000 Nano Assay on Agilent BioAnalyzer 2100 (Agilent Technologies, Santa Clara, CA). Commercially available high-density oligonucleotide, from Illumina whole genome gene expression BeadChips (Human HT12_V3_0_R3_11283641, Illumina Inc, San Diego, California, USA), were used with 48803 probes representing 37879 human transcripts. In brief, total RNAs were reverse transcribed to synthesize first- and second- strand complementary DNA (cDNA), followed by *in vitro* transcription to synthesize biotin-labeled complementary RNA (cRNA) using TotalPrep-96 RNA amplification kit (Ambion). A total of 750 ng of biotin-labeled cRNA from each sample was hybridised to the HT12 BeadChip (Illumina Inc., San Diego, CA) at 58°C for 18 hours. The hybridised BeadChips were washed and labeled with streptavidin-Cy3 and then scanned with Illumina BeadScan and images imported into GenomeStudioV2010.1 (Illumina Inc) for data extraction. Data were normalised and analysed using GeneSpring 11.5 (Agilent Technologies, Santa Clara, CA). Microarray data were normalised by percentile shift to the 75th percentile with the baseline set to the median of all samples. Genes with low expression (20% or less above background noise) were filtered out from further analysis. The data presented here is MIAME compliant.

### In Vivo OSM Administration

Recombinant murine OSM (R&D systems) was administered to 5-wk-old male C57/Bl6 mice by calvarial injections. Briefly, mice were injected daily with 0.2 µg mOSM or PBS over the left hemicalvaria for 5 consecutive days. Calcein (20 mg/kg) was injected intraperitoneally on days 1 and 14. Parietal bones were collected 24 hours after the last injection, fixed, embedded in methyl methacrylate resin and sections were analyzed by histomorphometry using the Osteomeasure software (Osteomeasure, Osteometrics). All animal procedures were conducted under Home Office project licence PPL70/7335.

### Statistical Analysis

Data are presented as the mean and standard error of the mean (S.E.M.) of at least three independent experiments performed in triplicate. Statistical analysis was performed on GraphPad Prism version 5 (GraphPad software, San Diego, CA) using one way analysis of variance (ANOVA) with Bonferroni’s correction for multiple comparisons or alternatively Dunnett’s post-test correction where multiple results were compared against a standard or control series. Significant results were depicted using asterisks, where *: P<0.05, **: P<0.01, ***: P<0.001. Genes with significantly altered expressions were identified by one way ANOVA with Benjamini-Hochberg multiple testing correction, with p<0.01. The entities satisfying the significance analysis were passed on for the fold change analysis. For a given gene its change in expression was calculated by dividing its average normalised intensity in treated samples from the respective control values. Significantly differentially expressed genes were identified with functional classifications, pathways analysis and gene set enrichment determined.

## Supporting Information

Figure S1 **Monocyte osteogenic effect is not mediated by monocyte-derived cytokines.** ALP activity of MSCs was measured after treatment with increasing concentrations of human recombinant IL1β in control and osteogenic media (A), or in the presence of neutralising antibody to inhibit endogenous IL1β in MSC-macrophage (Mφ) co-cultures (B). ALP activity of MSC was also measured after treatment with increasing concentrations of human recombinant TNFα (C) or IL8 (D), IL10 (E) and IL6 (G). Lysates (10 µg of protein) from MSCs treated with either human recombinant IL10 (F) or IL6 (I) at increasing concentrations or for increasing duration were subjected to WB for detection of pSTAT3 and STAT3. Graphs show means ± SEM of three independent experiments performed in triplicate. Blots are representative of three independent experiments performed. *p≤0.05 ***p≤0.001.
(TIF)

Figure S2 **Monocyte osteogenic effect is not mediated by BMPs or TGFβ.** MSC-macrophage (Mφ) co-cultures (ratio 1∶10) were established in the presence of neutralizing antibodies against BMP2/4, BMP3, BMP7 and TGFβ at 10 µg/ml as well as IgG1 and IgG2B isotype controls and ALP activity quantified after 7 days. Graphs show means ± SEM of three independent experiments performed in triplicate.
(TIF)

Table S1 **Supernatant from MSC alone cultures and 10∶1 monocyte/MSC co-culture were tested for the presence of BMP2, BMP4, BMP7 and TGFβ1 using individual, commercially available enzyme-linked immunosorbent assays (all from R&D systems, Abington, UK) according to manufacturer’s instructions.** Assay sensitivity was defined as the lowest standard for each assays’ standard curve and expressed as ‘less than’ value at, or beyond the limit of sensitivity.
(DOCX)
