# Supplementary figures and images for: Monocytes Induce STAT3 Activation in Human Mesenchymal Stem Cells to Promote Osteoblast Formation
Source: PLoS One. 2012 Jul 3;7(7):e39871. doi: 10.1371/journal.pone.0039871 (PMC3389003; doi:10.1371/journal.pone.0039871)

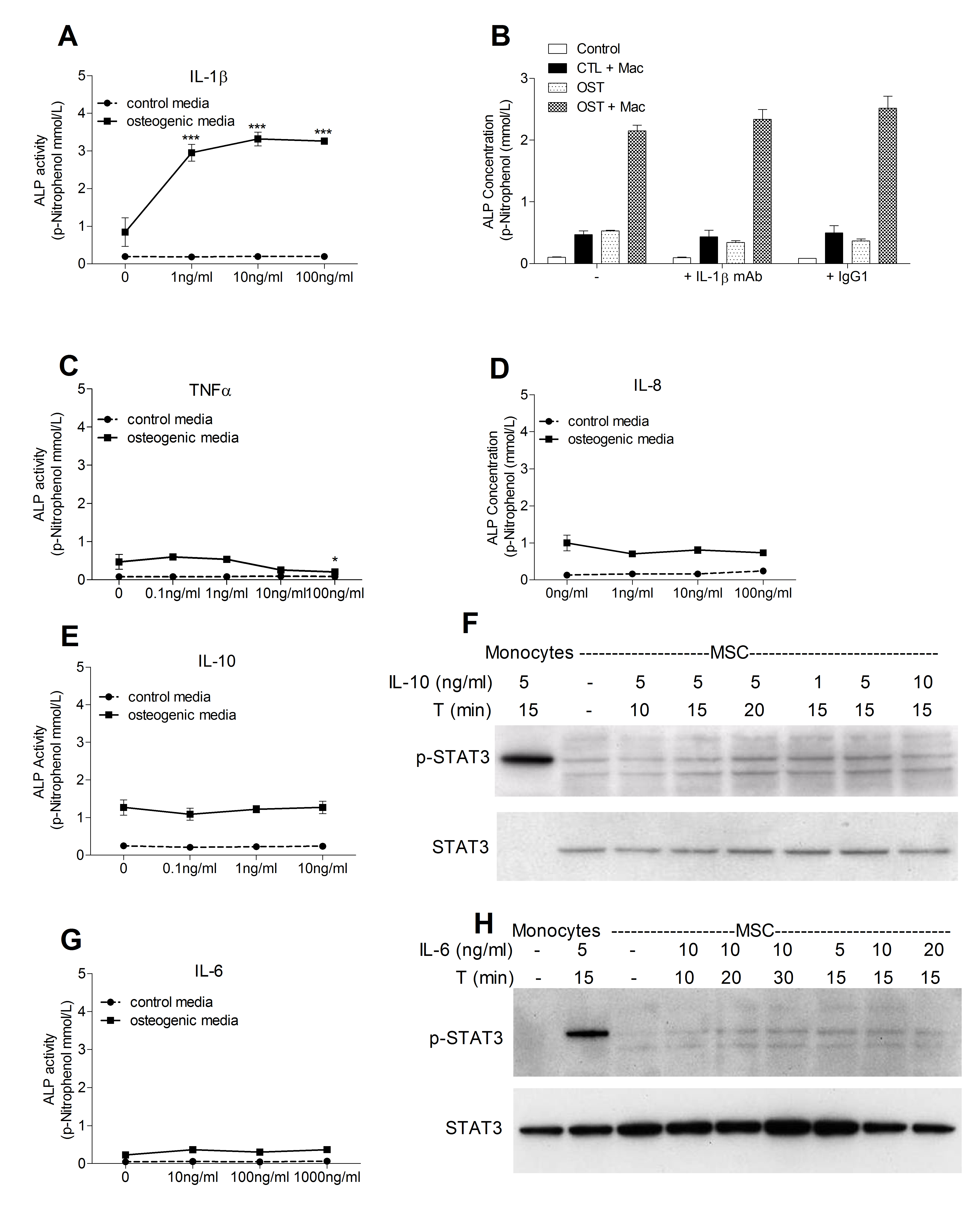

Supplement: Figure S1 — Monocyte osteogenic effect is not mediated by monocyte-derived cytokines. ALP activity of MSCs was measured after treatment with increasing concentrations of human recombinant IL1β in control and osteogenic media (A), or in the presence of neutralising antibody to inhibit endogenous IL1β in MSC-macrophage (Mφ) co-cultures (B). ALP activity of MSC was also measured after treatment with increasing concentrations of human recombinant TNFα (C) or IL8 (D), IL10 (E) and IL6 (G). Lysates (10 µg of protein) from MSCs treated with either human recombinant IL10 (F) or IL6 (I) at increasing concentrations or for increasing duration were subjected to WB for detection of pSTAT3 and STAT3. Graphs show means ± SEM of three independent experiments performed in triplicate. Blots are representative of three independent experiments performed. *p≤0.05 ***p≤0.001. (TIF) [file pone.0039871.s001.tif]

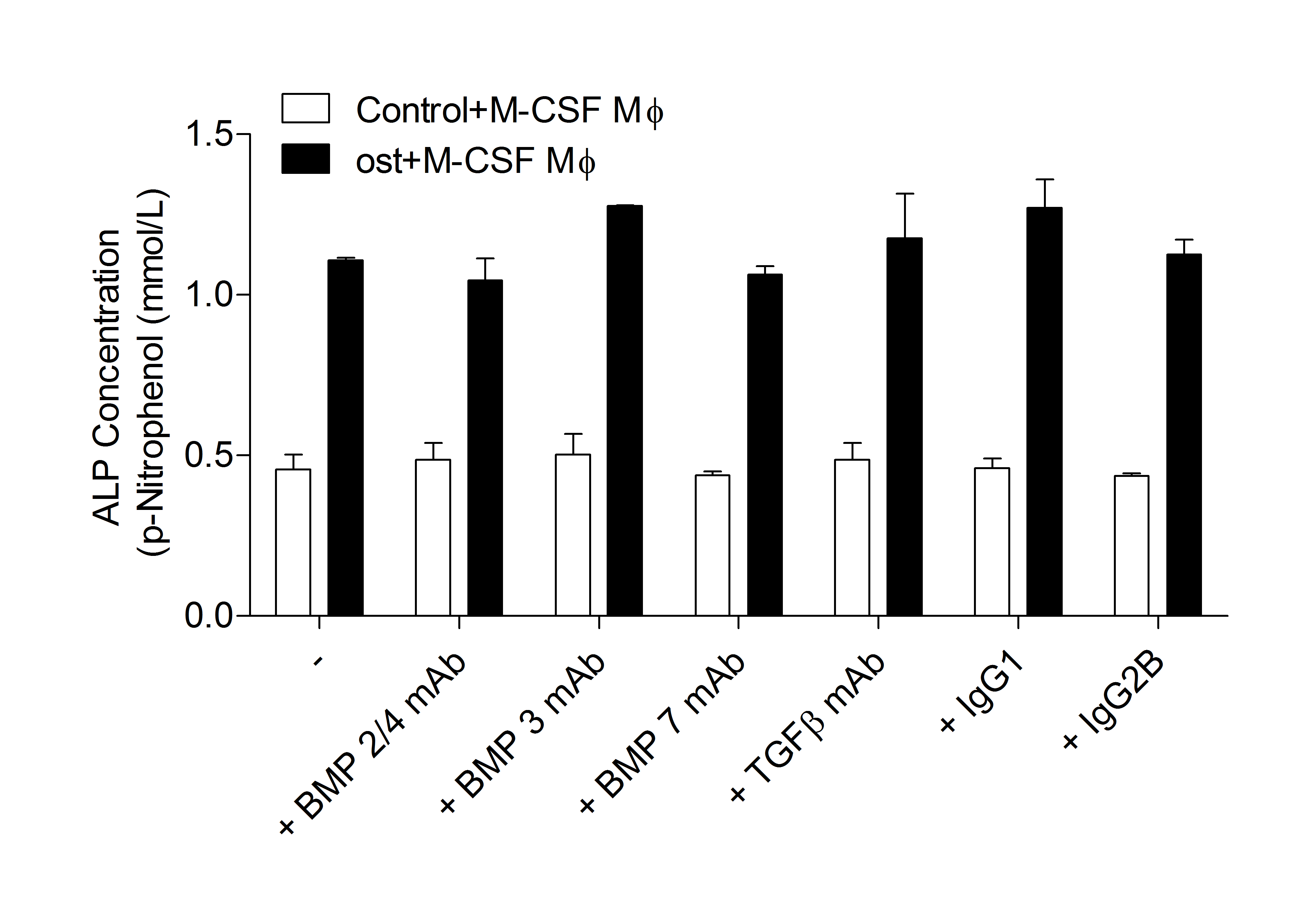

Supplement: Figure S2 — Monocyte osteogenic effect is not mediated by BMPs or TGFβ. MSC-macrophage (Mφ) co-cultures (ratio 1∶10) were established in the presence of neutralizing antibodies against BMP2/4, BMP3, BMP7 and TGFβ at 10 µg/ml as well as IgG1 and IgG2B isotype controls and ALP activity quantified after 7 days. Graphs show means ± SEM of three independent experiments performed in triplicate. (TIF) [file pone.0039871.s002.tif]
