# Supplementary material for: Monocytes Induce STAT3 Activation in Human Mesenchymal Stem Cells to Promote Osteoblast Formation
Source: PLoS One. 2012 Jul 3;7(7):e39871. doi: 10.1371/journal.pone.0039871 (PMC3389003; doi:10.1371/journal.pone.0039871)
Supplement: Table S1 — Supernatant from MSC alone cultures and 10∶1 monocyte/MSC co-culture were tested for the presence of BMP2, BMP4, BMP7 and TGFβ1 using individual, commercially available enzyme-linked immunosorbent assays (all from R&D systems, Abington, UK) according to manufacturer’s instructions. Assay sensitivity was defined as the lowest standard for each assays’ standard curve and expressed as ‘less than’ value at, or beyond the limit of sensitivity. (DOCX) [file pone.0039871.s003.docx]

**Table S1** BMP and TGFβ1 concentration in MSC and 10:1 monocyte/MSC culture supernatants

| Growth Factor | MSC alone | 10:1 monocytes:MSC |
| --- | --- | --- |
| BMP2 | 14.965 | 8.515 |
| BMP4 | 2.555 | 2.28 |
| BMP7 | <5 | <5 |
| TGFβ1 | 1049.26 | 957.235 |

**Table S1** Supernatant from MSC alone cultures and 10:1 monocyte/MSC co-culture were tested for the presence of BMP2, BMP4, BMP7 and TGFβ1 using individual, commercially available enzyme-linked immunosorbent assays (all from R&D systems, Abington, UK) according to manufacturer’s instructions. Assay sensitivity was defined as the lowest standard for each assays’ standard curve and expressed as ‘less than’ value at, or beyond the limit of sensitivity.
